# Ceftriaxone-Resistant *Neisseria gonorrhoeae*, Canada, 2017

**DOI:** 10.3201/eid2402.171756

**Published:** 2018-02

**Authors:** Brigitte Lefebvre, Irene Martin, Walter Demczuk, Lucie Deshaies, Stéphanie Michaud, Annie-Claude Labbé, Marie-Claude Beaudoin, Jean Longtin

**Affiliations:** Institut National de Santé Publique du Québec, Québec, Québec, Canada (B. Lefebvre, J. Longtin);; Public Health Agency of Canada, Winnipeg, Manitoba, Canada (I. Martin, W. Demczuk);; Centre Intégré Universitaire de Santé et de Services Sociaux de la Capitale-Nationale, Québec (L. Deshaies);; Direction de Santé Publique du Centre Intégré Universitaire de Santé et de Services Sociaux de la Capitale-Nationale, Québec (S. Michaud);; Université de Montréal, Québec (A.-C. Labbé);; Centre de Recherche en Infectiologie, Université Laval, Québec (M.-C. Beaudoin, J. Longtin)

**Keywords:** *Neisseria gonorrhoeae*, ceftriaxone-resistant, ceftriaxone, North America, Canada, bacteria, antimicrobial resistance, sexually transmitted infections, *penA*-60, mosaic *penA* allele, gonorrhea, antimicrobial susceptibility surveillance

## Abstract

We identified a ceftriaxone-resistant *Neisseria gonorrhoeae* isolate in a patient in Canada. This isolate carried the *penA*-60 allele, which differs substantially from its closest relative, mosaic *penA* XXVII (80% nucleotide identity). Epidemiologic and genomic data suggest spread from Asia. Antimicrobial susceptibility surveillance helps prevent spread of highly resistant *N. gonorrhoeae* strains.

Ceftriaxone is one of the last remaining treatments available for gonorrhea and a component of the recommended dual therapy with azithromycin in Canada (*1*). As of October 15, 2017, only 5 ceftriaxone-resistant *Neisseria gonorrhoeae* isolates had been reported worldwide (MIC range 0.5–2 mg/L) (*2*–*6*). The highest ceftriaxone MIC reported in Canada was 0.25 mg/L, representing only 0.45% (49/10,805) of all *N. gonorrhoeae* isolates tested during 2010‒2016 (*7*). We describe ceftriaxone-resistant *N. gonorrhoeae* isolated in Canada.

## The Study

An asymptomatic 23-year-old woman had a positive *N. gonorrhoeae* nucleic acid amplification test (NAAT) result (Cobas 4800 CTNG; Roche Diagnostics Canada, Laval, Canada) on January 17, 2017, obtained as part of a screening for sexually transmitted infections (STIs). Upon receiving the result, a physician instructed the patient to follow up with the STI clinic to have proper counselling. She visited on January 24 and obtained a prescription of single-dose cefixime 800 mg and azithromycin 1 g (recommended therapy according to Québec STI Treatment Guidelines) (*8*). Because the patient was from a low-prevalence population, the healthcare provider decided to perform a genital gonorrhea culture. The culture was positive for *N. gonorrhoeae* (no. GC063564/47707), thus confirming the positive NAAT result.

Because antimicrobial susceptibility testing (Etest, bioMérieux, Marcy l’Etoile, France) demonstrated nonsusceptibility of the isolate to ceftriaxone and cefixime but susceptibility to azithromycin, a second follow-up visit was requested by the practitioner. The second visit occurred February 7, 2017, and the patient was then prescribed empirically a single 2-g dose of azithromycin. Tests of cure by NAAT and cervical culture were performed during this visit and were negative for *N. gonorrhoeae*, indicating a successful initial treatment with cefixime and azithromycin administered 14 days earlier.

The patient reported a month-long sexual relationship 60 days before the STI screening. The partner was assessed by clinical examination and screening tests and treated with cefixime 800 mg and azithromycin 1 g on January 27, 2017. He was asymptomatic and his urinary NAAT screening result was negative for *N. gonorrhoeae*. He did not have sex with men or a sex worker but did report unprotected sexual activity during a trip to China and Thailand in November 2016, before his relationship with the patient in this case study. Information about antimicrobial drug use during his trip to Asia was not available. He was followed up in February 2017, and NAAT test results of his urine and pharyngeal specimens were again both negative for gonorrhea. Public health professionals also contacted the case study patient’s next-to-last partner (5 months earlier), and his screening result was also negative for *N. gonorrhoeae*.

The bacterial isolate from our patient was confirmed to be *N. gonorrhoeae* by API NH (bioMérieux), VITEK (bioMérieux), matrix-assisted laser desorption/ionization time-of-flight mass spectrometry (bioMérieux), and whole-genome sequencing. Antimicrobial susceptibilities for isolate GC063564 were confirmed by using the agar dilution method according to the Clinical and Laboratory Standards Institute protocol (*9*). The strain was resistant to ceftriaxone (MIC 1 mg/L), cefixime (MIC 2 mg/L), ciprofloxacin (MIC 32 mg/L), and tetracycline (MIC 4 mg/L) and susceptible to azithromycin (MIC 0.5 mg/L). The Clinical and Laboratory Standards Institute does not have a resistance breakpoint for cefixime or ceftriaxone but defines susceptibility at an MIC <0.25 mg/L. The European Committee on Antimicrobial Susceptibility Testing defines cefixime and ceftriaxone resistance as MIC >0.125 mg/L (*10*), and the World Health Organization defines decreased susceptibility to cefixime as an MIC ≥0.25 mg/L and to ceftriaxone as MIC ≥0.125 mg/L (*11*). Although the defined resistance breakpoint is not consistent among these organizations, a ceftriaxone MIC 1 mg/L has been previously reported as resistance (*2*,*6*).

We performed molecular typing in silico using whole-genome sequence data (BioProject PRJNA415047). We sequenced the isolate with the Illumina MiSeq platform (Illumina, San Diego, CA, USA) and used genomic quality, assembly, and annotation pipelines as previously described (*12*). The multilocus sequence type (ST) of GC063564 was ST1903, and *N. gonorrhoeae* multiantigen sequence type (MAST) was ST1614. Using a novel antimicrobial genomic sequence analytic tool called NG-STAR (*N. gonorrhoeae* Sequence Typing for Antimicrobial Resistance) (*13*), we identified the isolate GC063564 as NG-STAR ST233, which contains a mosaic *penA* allele, *mtrR*-35A deletion, *porB* G120K/A121D, *ponA* L421P, *gyrA* S91F/D94A, *parC* S87R, and no 23S rRNA A2059/C2611 mutations. The GC063564 isolate also had an *rpsJ* V57M mutation, and *tetM* was not detected.

The molecular antimicrobial resistance profile corresponds to the MICs determined phenotypically (*13*). The multilocus sequence type (ST1903) and mosaic *penA* allele (*penA*-60) of this isolate from Canada were identical to those of the ceftriaxone- and multidrug-resistant *N. gonorrhoeae* FC428 isolated in 2015 in Japan (*14*). *PenA*-60 has a mosaic penicillin-binding protein 2 structure with 2 key mutations (A311V and T483S) that confer ceftriaxone resistance. *PenA*-60 differs substantially from previously described *penA* types (*5*), resembling only 80% of the closest-related mosaic allele *penA*-XXVII. Bacterial isolates GC063564 and FC428 had identical *porB1b* NG-MAST alleles but different *tbpB* alleles (GC063564 had *tbpB*-33; FC428 had *tpbB*-21), resulting in different *N. gonorrhoeae* MAST profiles (ST-1614 for GC063564 and ST-3435 for FC428). The variation in the *N. gonorrhoeae* MAST types between the 2 isolates collected 2 years apart is not unexpected, considering the highly recombinant nature of the *N. gonorrhoeae* genome (*15*).

## Conclusions

We identified a ceftriaxone-resistant *N. gonorrhoeae* isolate in Canada that contained the *penA*-60 allele formerly reported in Japan in 2015. Epidemiologic information suggests international spread of a *penA* allele associated with high-level ceftriaxone resistance. Antimicrobial susceptibility surveillance successfully identified this novel isolate introduced into Canada and prompted public health officials to rapidly conduct an investigation to prevent further spread in the community. In an era of multidrug-resistant gonorrhea, ongoing antimicrobial susceptibility surveillance of *N. gonorrhoeae* is critical to support treatment guidelines, public health intervention, and protection.
